# Antimalarial artesunate–mefloquine versus praziquantel in African children with schistosomiasis: an open-label, randomized controlled trial

**DOI:** 10.1038/s41591-023-02719-4

**Published:** 2024-01-04

**Authors:** Emmanuel Bottieau, Moustapha Mbow, Isabel Brosius, Clémentine Roucher, Cheikh Tidiane Gueye, Ousmane Thiam Mbodj, Babacar Thiendella Faye, Annelies De Hondt, Bart Smekens, Diana Arango, Christophe Burm, Achilleas Tsoumanis, Linda Paredis, Yven Van Herrewege, Idzi Potters, Joachim Richter, Anna Rosanas-Urgell, Badara Cissé, Souleymane Mboup, Katja Polman

**Affiliations:** 1 Department of Clinical Sciences, Institute of Tropical Medicine, Antwerp, Belgium; 2 Institute for Health Research, Epidemiological Surveillance and Training (IRESSEF), Dakar, Senegal; 3 Department of Immunology, Cheikh Anta Diop University, Dakar, Senegal; 4 Department of Biomedical Sciences, Institute of Tropical Medicine, Antwerp, Belgium; 5 Institute of Tropical Medicine and International Health, Charité Universitätsmedizin, Berlin, Germany; 6 Department of Public Health, Institute of Tropical Medicine, Antwerp, Belgium; 7 Department of Health Sciences, Vrije Universiteit Amsterdam, Amsterdam, the Netherlands

**Keywords:** Parasitic infection, Randomized controlled trials

## Abstract

Schistosomiasis treatment entirely relies on a single drug, praziquantel, prompting research into alternative therapeutics. Here we evaluated the efficacy and safety of the antimalarial combination artesunate–mefloquine for the treatment of schistosomiasis in a proof-of-concept, pragmatic, open-label, randomized controlled trial in primary schools of six villages endemic for schistosomiasis in northern Senegal. Children (6–14 years) were eligible if *Schistosoma* eggs were detected by microscopy in urine and/or stool. In total, 726 children were randomized 1:1 to praziquantel (standard care: 40 mg kg^−1^ single dose; *n* = 364) or to artesunate–mefloquine (antimalarial dosage: artesunate 4 mg kg^−1^ and mefloquine 8 mg kg^−1^ daily for three consecutive days; *n* = 362). Eight children not meeting the inclusion criteria were excluded from efficacy analysis. Median age of the remaining 718 participants was 9 years; 399 (55.6%) were male, and 319 (44.4%) female; 99.3% were infected with *Schistosoma haematobium* and 15.2% with *S. mansoni*. Primary outcomes were cure rate, assessed by microscopy, and frequency of drug-related adverse effects of artesunate–mefloquine versus praziquantel at 4 weeks after treatment. Cure rate was 59.6% (208/349) in the artesunate–mefloquine arm versus 62.1% (211/340) in the praziquantel arm. The difference of −2.5% (95% confidence interval (CI) −9.8 to 4.8) met the predefined criteria of noninferiority (margin set at 10%). All drug-related adverse events were mild or moderate, and reported in 28/361 children receiving artesunate–mefloquine (7.8%; 95% CI 5.4 to 11.0) versus 8/363 (2.2%; 95% CI 1.1 to 4.3) receiving praziquantel (*P* < 0.001). Artesunate–mefloquine at antimalarial dosage was moderately safe and noninferior to standard-care praziquantel for the treatment of schistosomiasis, predominantly due to *S. haematobium*. Multicentric trials in different populations and epidemiological settings are needed to confirm these findings. ClinicalTrials.gov identifier: NCT03893097.

## Abstract (web summary)

In a proof-of-concept randomized trial in schoolchildren from Northern Senegal, artesunate–mefloquine at antimalarial dosage was noninferior to standard-care praziquantel for the treatment of schistosomiasis.

## Main

Schistosomiasis is a chronic helminth infection affecting more than 230 million individuals worldwide and causing major chronic morbidity^1^. About 90% of the burden lies with poor rural communities of sub-Saharan Africa, where *Schistosoma haematobium* and *Schistosoma mansoni* predominate^2^. These two species cause organ-specific pathology of the urogenital tract and hepato-intestinal system, respectively, as well as nonspecific morbidities such as anemia, malnutrition and growth impairment^3^. Currently, treatment of schistosomiasis relies on a single drug, praziquantel, which is active against adult worms of all *Schistosoma* species, simple to administer, safe, well tolerated and cheap^4–6^. The World Health Organization (WHO)-recommended, standard-care, 40 mg kg^−1^ single-dose praziquantel treatment reaches an aggregated parasitological cure rate of 75% for both predominant *Schistosoma* species^7,8^, and egg reduction rates above the 90% threshold set for satisfactory drug efficacy^9^. However, praziquantel has little activity on juvenile worms, and reinfections remain an unsolved challenge, especially in high-transmission areas such as sub-Saharan Africa^10^. In addition, the efficacy of praziquantel is jeopardized by its extensive and increasing use for preventive chemotherapy. This strategy is based on mass drug administration (MDA) of praziquantel at regular intervals to at-risk populations (mainly primary-school-aged children) and aims to reduce infection intensity and prevent the development of severe egg-related morbidity^6^. There is indeed some preliminary evidence of decreased susceptibility of schistosome worms in children with high exposure to MDA^11^.

Research on much-needed alternative treatments is progressing slowly^3,4^, and new compounds are still in preclinical evaluation^12^. Repurposed antimalarial drugs such as artemisinin derivatives and mefloquine have demonstrated in vitro activity against *Schistosoma* adult and/or juvenile worms^13,14^, but the clinical efficacy of each drug as monotherapy did not reach that of praziquantel^15–19^. An exploratory trial suggested, however, that combining artesunate and mefloquine could be as effective as praziquantel for the treatment of schistosomiasis^16^, but another one did not find consistent activity^20^. Artesunate–mefloquine exists as a fixed-dose combination for the treatment of malaria^21^, and is one of the well-established artemisinin-based combination therapies (ACTs) recommended by the WHO. So far, evidence of a potential clinical activity of artesunate–mefloquine against *Schistosoma* infection remains inconclusive, although the need of a backup treatment for praziquantel is pressing. Also, having an available drug therapy with dual effects on malaria and schistosomiasis could open attractive perspectives in the case management of coinfected individuals, as well as integrated chemoprevention for communities in the large co-endemic areas.

In this Article, we present the results of SchistoSAM, whose primary objectives were to compare the parasitological cure rate and safety of a 3-day course of the artesunate–mefloquine combination at antimalarial dosage versus the standard-care single-dose praziquantel for the treatment of schistosomiasis in African primary school-aged children^22^. The secondary objectives were (1) to determine the egg reduction rates by *Schistosoma* species, (2) to assess the cumulative antischistosomal efficacy and toxicity of one and two additional courses of artesunate–mefloquine, and (3) to monitor the incidence of clinical malaria and explore the frequency of *Plasmodium* *falciparum* infection and possible emergence of resistant markers.

Other components of the SchistoSAM study, aiming to assess the performance of novel schistosomiasis diagnostics as tools for monitoring treatment response, will be published elsewhere.

## Results

### Patient disposition

The trial took place in the primary schools of six selected villages (Yetty-Yone, Nder, Pakh, Gnith, Colona and Ronkh) in Richard Toll District, located in the northern Saint-Louis Region of Senegal. The area is co-endemic for *S. mansoni* and *S. haematobium*, with a reported prevalence of schistosomiasis of above 80% in school-aged children^23^. Malaria transmission is seasonal and annual incidence was 0.8/1,000 inhabitants in 2019 and in 2020 in this area^24^, below the threshold corresponding to very low malaria incidence (<1/1,000 inhabitants). Based on surveys in Senegalese school-aged children, the estimated prevalence of soil-transmitted helminthiasis is 8.2%, and 2–5% for hookworm infection^25,26^.

The selection of the six villages was made before the trial, based on a parasitological survey in primary schools that demonstrated high schistosomiasis prevalence. During this survey, 726 primary school-aged children were enrolled from a total of 818 who were screened by parasitological examination of one urine and one stool sample (Methods). Enrollments per village were as follow: 322/360 (89.4%) in Gnith; 170/196 (86.7%) in Ronkh; 86/90 (95.6%) in Nder; 72/75 (96%) in Yetti-Yone; 58/69 (84.1%) in Colona; and 18/28 (64.3%) in Pakh.

**Fig. 1:**
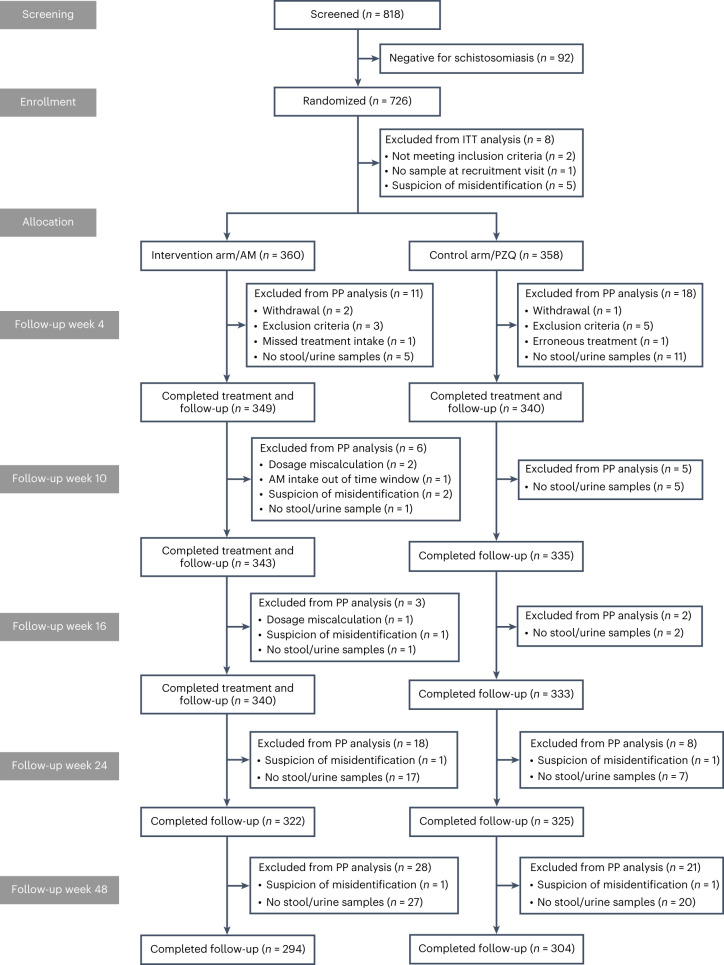
CONSORT flow diagram of the SchistoSAM trial and analysis population. AM, artesunate–mefloquine; PZQ, praziquantel; ITT, intention to treat; PP, per protocol.

These 726 children were enrolled and randomized at the baseline assessment (that is clinical, laboratory, parasitological and ultrasound assessment; Methods) from 29 November until 30 December 2019, but 8 children were eventually excluded from all efficacy analyses because they did not meet the inclusion criteria (Fig. 1).

The baseline characteristics of the remaining 718 participants were similar between trial arms as shown in Table 1. Median age was 9 years (interquartile range 7–11 years); 399 were male (55.6%) and 319 female (44.4%). Of all participants, 713 (99.3%) were infected with *S. haematobium*, alone (609; 84.8%) or coinfected with *S. mansoni* (104; 14.5%). The remaining participants (5; 0.7%) had *S. mansoni* mono-infection. Most participants were asymptomatic (615; 85.7%), with gross hematuria being reported by 65 (9.0%) children. Mean hemoglobin level was 12.0 mg dl^−1^ (95% confidence interval (CI) 11.3 to 12.7). Indirect markers of schistosomiasis morbidity were frequently found: microhematuria in 55.8% (369/661) and fecal occult blood in 24.3% (166/682) of the participants). In contrast, ultrasound abnormalities were infrequent, with urinary involvement in about 5% and liver pattern changes indicative of schistosomiasis fibrosis in less than 2% of the participants.

**Table 1:** Baseline characteristics of study participants in the intent-to-treat analysis (*n* = 718)

	Total (*n* = 718)	AM (*n* = 360)	PZQ (*n* = 358)
Male gender	399 (55.6)	197 (54.7)	202 (56.4)
Female gender	319 (44.4)	163 (45.3)	156 (43.6)
Age (years), median (IQR)	9.00 (7.00 to 11.0)	9.00 (7.00 to 11.0)	9.00 (7.00 to 11.0)
Height (cm), median (IQR)	132 (123 to 141)	133 (123 to 142)	131 (122 to 140)
Weight (kg), median (IQR)	25.0 (21.0 to 31.0)	25.0 (21.0 to 31.0)	25.0 (21.0 to 30.0)
*Schistosoma* species			
Single *S. haematobium* infection	609 (84.8)	306 (85.0)	303 (84.6)
Single *S. mansoni* infection	5 (0.70)	5 (1.39)	0 (0.00)
Mixed infection	104 (14.5)	49 (13.6)	55 (15.4)
Schistosomiasis infection intensity^a^			
*S. haematobium*			
Light (<50 eggs per 10 ml urine)	585 (81.5)	294 (81.7)	291 (81.3)
Heavy (>50 eggs per 10 ml urine)	128 (17.8)	61 (16.9)	67 (18.7)
*S. mansoni*			
Light (1–99 eggs per gram stool)	87 (12.1)	44 (12.2)	43 (12.0)
Moderate (100–399 eggs per gram stool)	19 (2.65)	9 (2.50)	10 (2.79)
Heavy (>400 eggs per gram stool)	3 (0.42)	1 (0.28)	2 (0.56)
Clinical symptoms			
None	615 (85.7)	307 (85.28)	308 (86.03)
Abdominal pain	16 (2.23)	11 (3.06)	5 (1.40)
Blood in stool	14 (1.95)	7 (1.94)	7 (1.96)
Painful micturition	8 (1.11)	4 (1.11)	4 (1.12)
Gross hematuria	65 (9.05)	31 (8.61)	34 (9.50)
Indirect morbidity markers			
Presence of anemia (hemoglobin level <11.5 g dl^−1^)	229 (31.9)	112 (31.1)	117 (32.7)
Hemoglobin level (g dl^−1^), median (IQR)	12.0 (11.3 to 12.7)	12.0 (11.3 to 12.8)	12.0 (11.3 to 12.7)
Presence of fecal occult blood (positive or trace)	166/682 (24.3)	79/338 (23.4)	87/344 (25.3)
Presence of microhematuria (1+ to 4+ on urine dipstick)	369/661 (55.8)	183/339 (54.0)	186/322 (57.8)
Presence of proteinuria (1+ to 3+ on urine dipstick)	294/661 (44.5)	146/339 (43.1)	148/322 (46.0)
Abdominal ultrasound findings			
Uni- and/or bilateral ureteral abnormalities (partial dilatation)	5/527 (1.0)	2/267 (0.8)	3/258 (1.2)
Focal/multifocal bladder abnormalities (wall irregularities or thickness, or masses >10 mm)	24/537 (4.5)	7/274 (2.6)	17/259 (6.6)
Liver abnormal patterns (C to F)^b^	9/531 (1.7)	4/271 (1.5)	5/260 (1.9)

^a^The intensity of infection for the microscopy results is categorized according to WHO recommendations^9^.

^b^Patterns C to F correspond to ultrasound images indicating various degrees of liver fibrosis attributable to schistosomiasis.

Note: all results are presented as *n*, or *n*/*n* (%), except otherwise mentioned; IQR denotes interquartile range.

### Primary outcomes

**Fig. 2:**
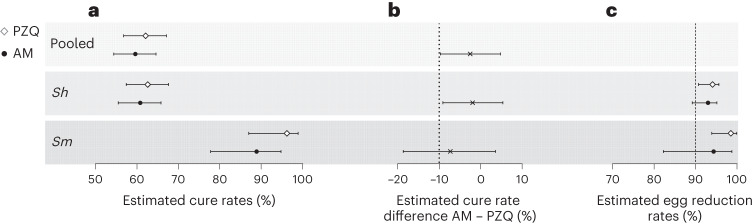
Parasitological efficacy 4 weeks post treatment. Efficacy results are shown as determined by microscopy for praziquantel (PZQ, white diamond) and artesunate–mefloquine (AM, black circle) in participants overall (pooled, *n* = 718) and infected by *S. haematobium* (*Sh*, *n* = 713) or *S. mansoni* (*Sm*, *n* = 109). **a**, Estimated cure rates (proportion of participants without any *Schistosoma* eggs at week 4) with 95% CI. **b**, Cure rate difference of artesunate–mefloquine compared with praziquantel with 95% CI. The dotted line represents the noninferiority margin for the difference in cure rates. **c**, Estimated egg reduction rates with 95% bootstrap CI. The striped line represents the efficacy threshold defined by WHO. AM, artesunate–mefloquine; PZQ, praziquantel.

Four weeks after treatment, 689 children (artesunate–mefloquine arm: 349; praziquantel arm: 340) were evaluated for parasitological efficacy; 29 participants were excluded from the per-protocol analysis (Fig. 1). As shown in Fig. 2 (and Supplementary Table 1), cure rate, as assessed by microscopy, in the artesunate–mefloquine arm was noninferior to that in the praziquantel arm (cure rate 59.6% (95% CI 54.4 to 64.6) versus 62.1% (95% CI 56.8 to 67.1); cure rate difference: −2.5% (95% CI −9.7 to 4.8), within the pre-established 10% margin of noninferiority; Methods). Per-species analysis revealed that artesunate–mefloquine was noninferior to praziquantel for *S. haematobium* infection (cure rate 60.8% (95% CI 55.5 to 65.8) versus 62.7% (95% CI 57.4 to 67.6), respectively; cure rate difference: −1.9% (95% CI −9.1 to 5.4)), but this could not be confirmed for the smaller number of children infected with *S. mansoni* (cure rate 88.9% (95% CI 77.8% to 94.8%) versus 96.2% (95% CI 87.0–98.9); cure rate difference: −7.3% (95% CI −18.7 to 3.6)).

For the safety analysis (Table 2), up to 4 weeks post-treatment, 28 drug-related adverse events (AEs) were reported in the 361 children who received at least one artesunate–mefloquine dose (7.8%; 95% CI 5.4 to 11.0), a higher frequency when compared with 8 occurrences in the 363 participants who got praziquantel (2.2%; 95% CI 1.1–4.3; *P* < 0.001). All artesunate–mefloquine-related AEs were mild or moderate and consisted mainly of abdominal pain and vomiting. Neurological AEs were rare and mild (Table 2).

**Table 2:** Frequency, severity, type and drug-relatedness of AE reports per study arm (‘as treated’ analysis)

	PZQ up to week 4 (*n* = 363)	AM up to week 4 (*n* = 361)	AM up to week 16 (*n* = 361)
Any AEs	14 (3.9; 2.3 to 6.4)^a^	29 (8.0; 5.6 to 11.3)^a^	41 (11.4; 8.5 to 15.0)
Any drug-related AEs	8 (2.2; 1.1 to 4.3)^a^	28 (7.8; 5.4 to 11.0)^a^	36 (10.0; 7.3 to 13.5)
Gastrointestinal disorders	8 (2.2)	26 (7.2)	31 (8.6)
Abdominal pain	5 (1.4)	15 (4.2)	18 (5.0)
Vomiting	4 (1.1)	12 (3.3)	14 (3.9)
Diarrhea	1 (0.3)	2 (0.6)	2 (0.6)
Odynophagia	–	–	1 (0.3)
Nervous system disorders	–	4 (1.1)	7 (1.9)
Headache	–	3 (0.8)	5 (1.4)
Vertigo	–	1 (0.3)	2 (0.6)
Other disorders			
Skin rash	–	1 (0.3)	1 (0.3)
Hematuria	–	1 (0.3)	1 (0.3)
Pyrexia	–	1 (0.3)	1 (0.3)
Cough	–	–	1 (0.3)
SAEs	–	–	–
Death	–	–	–
Confirmed malaria cases	–	–	–

Note 1: AM denotes artesunate–mefloquine; PZQ denotes praziquantel.

Note 2: results are reported as *n* (%; 95% CI). All AEs (*n* = 41) reported in the AM arm up to week 16 were classified as mild (*n* = 5) or moderate (*n* = 36). No grade 3 (‘severe’) AEs were reported.

^a^*P* values, determined with the Fischer’s exact test, were 0.019 and <0.001 for the comparisons between frequency of any AEs and any drug-related AEs, respectively, in the artesunate–mefloquine and praziquantel arms at week 4.

### Secondary outcomes

As reported in Table 3, the arithmetic egg reduction rates were 93.0% (95% CI 89.2 to 95.2) and 94.4% (95% CI 82.2 to 98.8) at week 4 after artesunate–mefloquine administration for *S. haematobium* and *S. mansoni*, respectively, with no statistical difference compared with those obtained with praziquantel (94.1% (95% CI 90.7 to 95.7) and 98.5% (95% CI 93.9 to 100), respectively). The geometric egg reduction rates was nearly equal between arms for *S. haematobium* (85%) and for *S. mansoni* (96%).

**Table 3:** Arithmetic and geometric egg reduction rates (ERRs in %) per *Schistosoma* species and study arm

Species	Visit	Mean	Artesunate–mefloquine ERR (95% CI)	Praziquantel ERR (95% CI)
*S. haematobium*	Week 4	Arithmetic	93.03 (89.22 to 95.19)	94.15 (90.74 to 95.67)
		Geometric	85.56 (83.41 to 87.6)	85.53 (83.28 to 87.56)
	Week 10	Arithmetic	95.53 (91.5 to 97.19)	91.51 (86.03 to 94.99)
		Geometric	87.65 (85.85 to 89.33)	87.09 (84.86 to 88.98)
	Week 16	Arithmetic	97.74 (95.01 to 98.85)	93.44 (87.57 to 96.41)
		Geometric	89.05 (87.24 to 90.59)	87.97 (85.95 to 89.74)
	Week 24	Arithmetic	98.6 (97.43 to 99.15)	94 (88.33 to 96.63)
		Geometric	89.14 (87.24 to 90.59)	87.53 (85.45 to 89.37)
	Week 48	Arithmetic	33.15 (12.55 to 49.63)	31.52 (10.41 to 49.28)
		Geometric	45.61 (32.94 to 55.52)	36.5 (21.35 to 49.08)
*S. mansoni*	Week 4	Arithmetic	94.38 (82.25 to 98.79)	98.55 (93.93 to 100)
		Geometric	96.13 (93.93 to 97.4)	96.97 (95.76 to 97.78)
	Week 10	Arithmetic	98.59 (95.11 to 99.75)	98.26 (85.21 to 100)
		Geometric	96.85 (95.81 to 97.76)	96.96 (95.72 to 97.78)
	Week 16	Arithmetic	99.53 (96.58 to 100)	99.42 (96.44 to 100)
		Geometric	97.33 (96.45 to 98.08)	97.14 (96.22 to 97.95)
	Week 24	Arithmetic	89.07 (69.25 to 97.61)	97.1 (91.35 to 99.06)
		Geometric	95.79 (93.68 to 97.17)	96.46 (95.25 to 97.41)
	Week 48	Arithmetic	36.79 (−28.58 to 80.02)	52.34 (14.8 to 76.01)
		Geometric	85.12 (73.57 to 91.65)	89.23 (80.67 to 93.85)

Note: ERR denotes egg reduction rate (%).

Determination of egg reduction rates is based on the WHO recommendations^9^.

The number of eggs recorded in two urine (10 ml each) and two stool samples (two slides of 25 mg for each stool sample) were adjusted to number of eggs per 10 ml for urine samples and per gram for stool samples.

The eggs in urine samples were calculated as: number of eggs per 10 ml = 10 × (number of eggs in sample 1 + number of eggs in sample 2)/(volume of sample 1 + volume of sample 2).

The number of eggs in stool samples was calculated as: number of eggs per gram = 1,000 × (sum of eggs in all available slides)/(25 × number of slides).

Of note, ectopic eggs were not taken into consideration in the calculations.

The arithmetic egg reduction rate (ERR) was calculated using the following formula: ERR_*i*_ = 100 × (1 − (mean egg counts at visit *i*)/(mean egg counts at baseline)). For the geometric ERR, the used formula was exp(mean(log(*x* + 1)) − 1), to include all samples with zero eggs, as described in Olliaro^38^. The CIs for the ERR were calculated using a bootstrap resampling method (with replacement) over 2,000 replicates and expressed as adjusted bootstrap 2.5th and 97.5th percentiles.

**Fig. 3:**
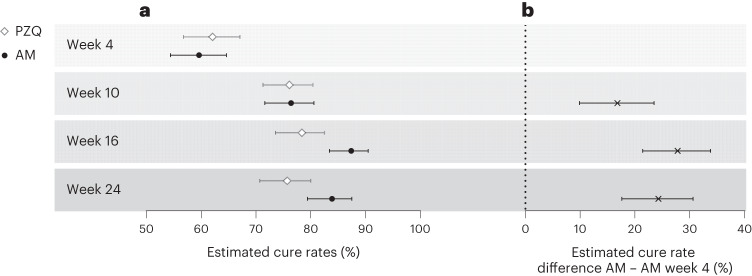
Parasitological efficacy of repeated administrations of artesunate–mefloquine. **a**, Cure rates (proportion of participants without any *Schistosoma* eggs) with 95% CI are shown as determined by microscopy in the praziquantel (PZQ, white diamond) and artesunate–mefloquine (AM, black circle) arms in participants infected with any *Schistosoma* species (*n* = 718). **b**, Cure rate difference with 95% CI of repeated administrations of artesunate–mefloquine (as assessed at weeks 10, 16 and 24 post-initial treatment), compared with week 4 assessment. Detailed numbers are available in Supplementary Table 2.

The cure rate, 4 weeks after the second and third administration of artesunate–mefloquine, is also reported in Fig. 3 and Supplementary Table 2. It increased from 59.6% (95% CI 54.4 to 64.6) after the first course to 76.4% (95% CI 71.6 to 80.6; cure rate difference week 10 − week 4: 16.8% (95% CI 9.9 to 23.5)) after the second dose, and to 87.4% (95% CI 83.4–90.5; cure rate difference week 16 − week 4: 27.8% (95% CI 21.4 to 33.8)) after the third dose. Increase of cure rate was observed for both species, but was more pronounced for *S. haematobium* infection (Fig. 3). Of note, at week 16 and week 24, cure rate was higher in the repeated artesunate–mefloquine arm (87.4% and 83.9%, respectively) than in the single-dose praziquantel arm (78.4% and 75.7%, respectively) (Supplementary Table 2). In addition to the 28 drug-related AEs reported within 4 weeks after the first artesunate–mefloquine course, only 8 new AEs were attributed to the second or third courses and all were reported as mild or moderate (Table 2). The rate of drug-related AEs increased from an initial 7.8% (up to week 4) to 10% in total for all three courses (up to week 16), with no notable change in the pattern of symptom. Of note, 80% of all reported AEs occurred within 1 week after each drug administration. Median duration of artesunate–mefloquine-related AEs was 3 days compared with 2.5 days for praziquantel. No serious or severe AEs were reported during the three consecutive courses of artesunate–mefloquine.

At the interim assessment (week 24; Methods), the frequency of reported gross hematuria substantially decreased to <1% (2/679) in the study population (compared with 9% in the baseline assessment). Similarly, the proportion of participants with microhematuria sharply decreased from 55.8% at baseline to 7.3% at week 24, while the reduction of fecal occult blood positivity was less striking (from 24.3% at baseline to 18.0% at week 24). No statistical difference was observed between arms at week 24 in the frequency of schistosomiasis-related symptoms and laboratory abnormalities. The scheduled ultrasound assessments at weeks 24 and 48 could not take place because of the coronavirus disease 2019 (COVID-19) crisis.

No participant developed clinical malaria during the 1-year observation period and no cases of *P. falciparum* malaria infection was retrospectively detected in either arm. On a side note, at week 48 (end of study), the proportion of children positive for *Schistosoma* spp. by microscopy was above 80% in both study arms. All children enrolled in the trial received standard-care praziquantel treatment at that moment, according to the protocol.

### Sensitivity analysis

As shown in Supplementary Table 1, noninferiority of a single course of artesunate–mefloquine compared with a single dose of praziquantel was also consistently proven for all sensitivity analyses in the intention-to-treat population.

## Discussion

In this proof-of-concept, pragmatic, open-label, randomized, noninferiority trial, we demonstrated that a 3-day course of artesunate–mefloquine at antimalarial dosage was noninferior to a standard single-dose of praziquantel, for the treatment of schistosomiasis in African schoolchildren. This observation was mainly driven by the effect on *S. haematobium* infection. The mean arithmetic egg reduction rates were, however, above the recommended threshold of 90% (ref. ^9^) for both *S. hematobium* and *S. mansoni* species. Drug-related AEs were more frequent in the artesunate–mefloquine arm compared with the praziquantel arm, but they were all mild or moderate. An additional second and third courses of artesunate–mefloquine substantially increased the cure rate compared with a single course, with only a marginal increment of AEs. The frequency of schistosomiasis symptoms and abnormal morbidity markers 24 weeks after initial treatment decreased in similar proportions in both study arms.

This is an adequately powered trial that evaluated the parasitological and clinical efficacy of the antimalarial combination artesunate–mefloquine as an alternative to praziquantel for the treatment of schistosomiasis. This study also explored the incremental benefit, as well as cumulative toxicity, of repeated courses of artesunate–mefloquine. Apart from its large sample size, other strengths of this trial included its pragmatic school-based design, which is similar to the MDA reality, the rigorous parasitological evaluation by experienced microscopists blinded to treatment, the careful follow-up of potential mefloquine toxicity up to 1 month after each drug administration, and the molecular monitoring of malaria infection on dried blood spots in both study arms. There were also some limitations. For the efficacy outcome, cure rates were assessed by conventional microscopy, which is still the WHO established standard for diagnosis of schistosomiasis and assessment of treatment response^9^. However, microscopy is considered an insensitive method that tends to overestimate treatment efficacy. Concurrently with microscopic examinations, we therefore evaluated a set of highly sensitive antigen-based (that is, circulating cathodic and anodic antigens) and DNA-based detection assays as alternative tests of cure^22^; due to space limitations, comparative results on the performance of those diagnostics will be published elsewhere. The use of the egg reduction rate as measure for the efficacy outcome would have substantially inflated the required sample size, beyond the scope of a proof-of-concept trial. In addition, the study design did not allow to investigate the parasitic efficacy of a single course of artesunate–mefloquine beyond the week 4 assessment, although activity of mefloquine on juvenile worms may provide longer protection than praziquantel. For the safety analysis, the absence of praziquantel placebo comparison at weeks 6 and 12 might have overestimated the difference in occurrence of AEs between the two treatment arms. Also, this proof-of-concept trial, deliberately conducted in an area with low malaria endemicity to focus on schistosomiasis endpoints, did not provide insights on the potential benefits and risks of single and repeated artesunate–mefloquine administrations on malaria infection or on the potential emergence of resistance. Finally, the ultrasound assessments scheduled at the interim (week 24) and final (week 48) assessments could not take place because of the COVID-19 pandemic, during which schools were closed and large gatherings forbidden. The frequency of ultrasound abnormalities was, however, rather low at the initial assessment. The sampling of urine, stool and blood was able to be kept on schedule through door-to-door visits throughout the study period.

The clinical efficacy of artemisinin derivatives on schistosomiasis has been found inferior to that of praziquantel^18,27^, as their activity is mainly restricted to the juvenile worms. In addition, the use of artemisinin derivatives in monotherapy as antischistosomal agents cannot be envisaged in the large areas of sub-Saharan Africa where *P. falciparum* is co-endemic, given the risk of malaria resistance to this key compound. Mefloquine is an antimalarial drug with activity against both juvenile and adult *Schistosoma* worms, but the clinical evidence of efficacy is very limited^16,19,20^. The evaluation of the artesunate–mefloquine combination has provided equivocal results in two small exploratory trials, so far^16,20^. The current trial demonstrates substantial antiparasitic activity of artesunate–mefloquine, with clinical benefit on schistosomiasis-related symptoms and morbidity, and the incremental effect of successive treatment courses. Additionally, its safety profile is confirmed, even in repeated administration, at least in the pediatric population^21,28,29^. Therefore, the important potential of artesunate–mefloquine as repurposed antischistosomal drug is highlighted, with the key advantage that it is a cheap and immediately available treatment. The slightly lower tolerance and need of 3-day administration would, however, position it as second-line treatment, in case of intolerance or decreased susceptibility to praziquantel, pending future feasibility and acceptability studies. Acute schistosomiasis in nonimmune travelers could be another clinical scenario where the artesunate–mefloquine combination, with its activity on juvenile worms, would be worth being evaluated against praziquantel.

Moreover, the concomitant antimalarial and antischistosomal activity of artesunate–mefloquine opens exciting research perspectives in coinfected patients and in co-endemic areas^30^. The dual benefit for coinfected patients appears quite obvious, but the simultaneous diagnosis of both infections is probably infrequent in first-line health facilities. However, artesunate–mefloquine could be studied as the preferred ACT in any child presenting with clinical malaria in regions moderately or highly prevalent for schistosomiasis. In a larger perspective, as transmission of both malaria and schistosomiasis peaks during the rainy season in Africa, it would also be interesting to explore the dual protective effect of seasonal administration of artesunate–mefloquine at the community level, by analogy with the seasonal preventive chemotherapies recommended by the WHO for malaria control^31–34^. Since repeated courses are acceptably safe and provide cumulative efficacy on schistosomiasis, it would be worth evaluating different schedules and timing of artesunate–mefloquine as seasonal chemoprevention against both malaria^31^ and schistosomiasis in school-aged children. In a next step, research into artesunate–mefloquine as an alternative seasonal (malaria) chemoprevention in children <5 years could also be considered, as this age group is also particularly affected by both conditions.

Perennial or seasonal intermittent preventive treatment in school-aged children is conditionally recommended by the WHO in malaria-endemic settings with moderate to high transmission, preferably with regimens not used locally as first or second-line malaria treatment^31^. Artesunate–mefloquine could be a good investigational candidate for seasonal intermittent preventive treatment in children of different age groups, in settings where prevalence of both malaria and schistosomiasis is moderate or high. However, the emergence of artemisinin resistance in the past few years in East Africa^35^ has increased legitimate concerns about the use of ACT-based preventive chemotherapies. Although there is no evidence that such interventions promote clinical resistance^36^, adequate molecular monitoring should be integrated into each new trial and routinely established wherever implementation of such strategy is being considered. Next-generation sequencing tools such as deep amplicon sequencing, capable of targeting multiple markers in a single assay, are gradually being deployed to support the surveillance activities of malaria control programs^37^.

In conclusion, the combination artesunate–mefloquine at antimalarial dosage is safe and its efficacy against schistosomiasis is noninferior to that of standard-care praziquantel, at least for *S. haematobium* infection. If similar results are reproduced in other epidemiological settings, especially where *S. mansoni* is more prevalent, or in other demographics, such as malaria-coinfected patients, more advanced schistosomiasis cases or pre-school-aged children, it could become one of the much-needed alternative drugs for individualized treatments.

## Methods

### Study design and setting

This was a proof-of-concept, pragmatic (school-based), open-label, phase 2b, randomized, active control, noninferiority trial, comparing the investigational treatment (3-day course of artesunate–mefloquine combination at antimalarial dosage) to the standard of care (standard 40 mg kg^−1^ single dose of praziquantel) for the treatment of schistosomiasis. The trial took place in the primary schools of six villages (Yetty-Yone, Nder, Pakh, Gnith, Colona and Ronkh), selected on the basis of a parasitological survey before the trial, in the Richard Toll District, located in the northern Saint-Louis Region of Senegal. This area is characterized by a high co-endemicity of both *S. haematobium* and *S. mansoni* species, and a very low malaria incidence.

### Participants

Participants for this trial were recruited among primary school-aged children (6–14 years old) from the six selected villages. The purpose and methodology of the trial were presented in detail to the community leaders, school directors, teaching staff and children’s parents. Written informed consent for schistosomiasis screening and trial participation upon positivity was asked by experienced study nurses from the children’s parents or legal guardians. Participants’ gender was assessed by parental reporting (for the younger children) and/or by self-reporting (for the older ones). Oral assent was also obtained from the children.

During the initial screening survey in the schools of the selected villages, all consenting children provided one urine and one stool sample for parasitological analyses in the laboratory of the Richard-Toll Hospital. Children fulfilling the inclusion criteria during this screening (that is, signed informed consent and presence of at least one egg of *S. haematobium* or *S. mansoni* in any of the urine or stool samples) were invited for a baseline assessment before enrollment in the trial.

Baseline assessment included parasitological confirmation and quantification of *Schistosoma* infection (both with microscopy and novel diagnostics, see below), as well as a medical and laboratory evaluation. Detailed descriptions were published elsewhere^22^. For the parasitological baseline assessment, two urine and two stool samples were collected on consecutive days. From each patient, 10 ml of each urine sample was filtered with the standard filtration technique, while a total of 50 mg (2 × 25 mg per slide) from each of both stool samples were examined using the duplicate Kato-Katz method. The resulting slides were examined microscopically (AML120EB biological microscope, a.m.l. sprl). Each of the two study microscopists read half of the urine and stool slides, and 10% of the slides we re-read by the other one every day. In case of discordant results (this occurred in less than 2% of the revised slides), those assessed by the most experienced microscopist were entered in the database. In accordance with the SchisoSAM protocol^22^, in parallel to conventional microscopy, DNA- and antigen-based assays (both the point-of-care circulating cathodic antigen and circulating anodic antigen) were also systematically run either in the study hospital or in reference laboratories (methods and results to be reported in a second manuscript). The medical evaluation was performed in the study schools and included the measurement of body weight and height, a clinical examination focused on schistosomiasis-related complications, the determination of the hemoglobin level via a finger prick (with HemoCue Hb 301, HemoCue), and an abdominal ultrasound exploration of urinary or hepatic abnormalities^39^ by an experienced radiologist with a portable machine (Samsung Medison SonoAce R3). The laboratory evaluation included the search for microhematuria in urine (with the heme reagent dipstick test Medi-Test Combi-5, Macherey-Nagel) and fecal occult blood in stool samples (with Mission test, Acon Laboratories) in the hospital laboratory, as indirect markers of schistosomiasis morbidity^40^. Informed consent was confirmed and validated by the study physician after a thorough check for exclusion criteria. The exclusion criteria were: (1) past or present diagnosis of epilepsy or psychiatric illness; (2) history of hypersensitivity to one of the study drugs (praziquantel or artesunate–mefloquine); (3) chronic medication for any reason; (4) exposure to praziquantel or any ACT within 3 months before inclusion; (5) current febrile illness or clinical malaria at the time of inclusion; (6) any severe underlying illness based on clinical judgment (for example, severe malnutrition), severe anemia defined by an hemoglobin level below 7 g dl^−1^, or severe chronic schistosomiasis, as assessed by clinical and/or ultrasound examination (7) planned travel for more than 1 month within the first 4 months after enrollment. Infected children not eligible for the study because of the presence of any exclusion criteria were administered a standard single-dose of praziquantel as standard of care, and referred to medical attention if necessary.

### Randomization and masking

A block randomization schedule, stratified by the two *Schistosoma* species, was prepared by the sponsor biostatistician, using SAS v9.4 (SAS Institute). In case of coinfection, patients were stratified within the *S. mansoni* group, as numbers were expected to be lower for this species. The individual randomization numbers and corresponding treatment arm were stored in sealed opaque envelopes and opened by the study physician for each child at enrollment. The participants and the clinical team were not blinded to treatment and clinical evolution, but the laboratory technicians and microscopists were.

### Procedures

Assenting eligible children were randomly allocated in a 1:1 ratio to standard of care (praziquantel) or the investigational treatment (artesunate–mefloquine). Standard of care consisted of oral administration of a single dose of praziquantel at the WHO-recommended dosage of 40 mg kg^−1^. The investigational drug combination was given orally at the antimalarial dosage of 4 mg kg^−1^ per day of artesunate and 8 mg kg^−1^ per day of mefloquine daily over three consecutive days (total dose of 12 mg kg^−1^ artesunate + 24 mg kg^−1^ mefloquine). Both praziquantel and artesunate–mefloquine were donated by the Indian manufacturer Cipla (prequalification WHO reference number: artesunate–mefloquine 25/50 MA078, artesunate–mefloquine 100/200 MA079, praziquantel NT003), which had not been involved in the development of this study protocol. After calculation of the adequate weight-based dosage, ingestion of both standard-of-care and investigational treatment was directly observed by the study staff in the schools, after which all children received a light meal. Each participant was monitored during 2 h for any adverse reactions. If a child vomited within half an hour, the full dose was repeated; if vomiting occurred after 30 min, but within 2 h, half of the dose was re-administered. If repeating the dose resulted in recurrent vomiting, treatment was discontinued.

In the artesunate–mefloquine arm, a second and third 3-day course of artesunate–mefloquine were administered under medical supervision 6 and 12 weeks after the initial treatment, respectively. A 6-week interval between each artesunate–mefloquine administration was chosen to reduce the risk of cumulative mefloquine-related neuropsychiatric AEs. Safety visits to the sites by physicians took place every day during the 3-day period of artesunate–mefloquine administration. Participants were also visited by study nurses one week after each drug administration to capture any additional AE. Medical, laboratory and parasitological follow-up assessments took place for all participants in both arms at week 4, 10, 16 (corresponding to 4 weeks after each artesunate–mefloquine course), as well as week 24 after initial treatment (interim assessment) and week 48 (final/end of study assessment). The follow-up sampling was organized within the primary schools in close collaboration with community health workers and teachers. All samples were cryopreserved to perform additional molecular and antigenic diagnostic investigations as detailed in the published study protocol^22^.

For the molecular diagnosis of malaria by quantitative polymerase chain reaction (qPCR), filter papers with dried blood samples (finger prick) from peripheral blood were punched and three circles of 5 mm in diameter was used for DNA extraction with QIAamp 96 DNA blood kit (Qiagen) in the research laboratory of the Unit of Malariology (ITM). Extracted DNA was eluted in 150 μl of water. Five microliters of DNA were used for qPCR analysis targeting *P. falciparum* var gene acidic terminal sequence (varATS, ~59 copies per genome) as previously described^41^. The limit of detection in our laboratory was 0.1 parasite μl^−1^. In case of positive *P. falciparum* samples, and to identify genetic variants associated with resistance, it was planned to perform a highly multiplexed deep sequencing assay (Pf AmpliSeq), which allows high-accuracy sequencing with higher coverage and lower cost than whole-genome sequencing^42^.

All participants, whether or not they completed the study, were offered praziquantel as standard of care at the final study visit (week 48 post-inclusion).

### Outcomes

The primary outcome was the cure rate as assessed by microscopy of urine and stool 4 weeks after treatment. Cure rate was defined as the proportion of egg-positive children at baseline, who became egg-negative 4 weeks after treatment in all collected samples. The co-primary outcome was the frequency and type of AEs and serious AEs (SAEs) in both arms. Any clinical sign or symptom was reported as an AE if it occurred or worsened after the start of study treatment. AEs were assessed in both arms by the study doctors during three days after treatment administration, and recorded by the study nurses and assisting community workers at weeks 1 and 4 post-treatment. Assessment of AEs was repeated following the same schedule in the artesunate–mefloquine arm only after the second and third courses. Nurses of the health centers in the six villages were also involved in the trial follow-up and instructed to contact the study physicians in case of any incidental problem occurring to a study participant between the visits of the study team.

Secondary outcomes included (1) the cure rates by *Schistosoma* species and the arithmetic and geometric egg reduction rates in both arms, at week 4 post-treatment, (2) the cumulative cure rates in the artesunate–mefloquine arm 10, 16 and 24 weeks after the initial treatment, (3) the cumulative frequency and type of AEs after each additional artesunate–mefloquine course, and (4) the number of episodes of clinical malaria among participants as captured by the regional surveillance system in place, as well as the prevalence of *P. falciparum* malaria infection in both study arms, as retrospectively assessed by quantitative polymerase chain reaction on dried blood spot samples. Of note, egg reduction rate was not retained as primary endpoint, because the sample size necessary to demonstrate noninferiority would have been much higher, beyond reach of a proof-of-concept trial.

Clinical, ultrasound, laboratory and parasitological data at baseline and during follow-up assessments were entered into Research Electronic Data Capture (REDCap, v8.10.4), an International Council for Harmonization-Good Clinical Practice compliant data capture system. The data system included password protection and internal validation checks to identify data that appeared inconsistent, incomplete or inaccurate.

### Statistical analysis

The sample size calculation was based on the hypothesis that the cure rate (as assessed by microscopy) after the first course of artesunate–mefloquine (that is, primary outcome) was noninferior to that of praziquantel with a power of 80%. Compared with the usual cure rate with standard single-dose praziquantel (estimated at 75% (95% CI 63 to 81)), a maximum difference of 10% was considered as an acceptable margin for noninferiority of artesunate–mefloquine. This large margin was clinically chosen considering the concomitant beneficial impact on malaria infection that could be expected with artesunate–mefloquine in the large co-endemic areas co-endemic settings. It was agreed upon among clinicians that this assumed additional benefit on malaria could justify a treatment with a slightly lower efficacy on schistosomiasis (compared with praziquantel). To confirm noninferiority at the chosen cutoff, the required sample size was 300 schoolchildren per arm, but to account for a 20% loss to follow-up, the total number of children to be randomized was 720 (360 per arm). For the efficacy analysis, both an intention-to-treat and a per-protocol approach were adopted, with the per-protocol analysis being used for the primary outcome, as recommended for noninferiority studies in the Consort statement on noninferiority trials (CONSORT—Reporting of Non-inferiority and Equivalence Randomized Trials: www.consort-statement.org) and International Council for Harmonization guidelines. The primary hypothesis for noninferiority was assessed by calculating the two-sided 95% Wilson CI for the difference in cure rates between arms (artesunate–mefloquine and praziquantel). If the resulting 95% CI was entirely above −10%, then noninferiority of the artesunate–mefloquine treatment could be concluded. For the safety evaluation, all participants who received at least one dose of study medication were included in the ‘as treated’ analysis. Safety events were described using patient counts and percentages with 95% CIs, and comparisons between arms (at week 4 post-treatment) and between artesunate–mefloquine courses were performed using Fisher’s exact test.

Statistical analysis were performed with R v4.2.2. No specific gender-based analysis was performed, as it was not considered as relevant for the trial on drug efficacy and safety.

### Ethics and inclusion statement

This project was designed through a longstanding partnership between the Institute of Tropical Medicine (ITM) in Antwerp, Belgium and the ‘Institut de Recherche en Santé de Surveillance Epidémiologique et de Formation’ (IRESSEF) in Dakar, Senegal. Since the early 2000s, multiple epidemiological and diagnostic studies on schistosomiasis have been conducted in connection with this scientific collaboration, especially in the Richard-Toll District, Saint-Louis Region of Senegal, as documented by a number of joint publications^43–50^. During the preparation of the project, other key players such as the National Control Programs (for Neglected Tropical Diseases and for Malaria) were consulted and informed about the study objectives, and their respective feedback was integrated in the final protocol. Roles and responsibilities were shared between Senegalese and Belgian researchers, with co-principal investigators and co-primary authors of each nationality.

The District of Richard-Toll was selected as trial site, for the epidemiological reasons explained in this manuscript, but also because an experienced field team (nurses, microscopists and laboratory technicians) has been present for about 20 years. This team, deeply rooted in the local communities, has carried out numerous schistosomiasis research activities over the years, often door to door, and has gained the full trust of village leaders and communities. As described in the published protocol^22^, the preparation of this community trial consisted of repeated dedicated visits to the population (parents, children, school principals, teaching staff and community leaders) of potentially eligible villages, to explain the objectives and methods, as well as the expected benefits and risks. Sufficient time was spent on questions and answers about the project during successive meetings. The engagement of the study team was instrumental in limiting the number of participants lost to follow-up and in ensuring field activities continued during the COVID-19 crisis.

All IRESSEF, ITM and local investigators collaborated on data ownership and authorship of publications related to the project. All analyses and results will be shared to national, regional and local co-investigators and stakeholders during a closing visit for this trial in Dakar and Richard-Toll.

### Ethics approval

The trial was approved by the Institutional Review Board of the Institute of Tropical Medicine (on 30 January 2019, Ref. 1269/18) and the Ethics Committee of the University of Antwerp, (on 21 January 2019, Ref. 19/02/005), in Antwerp, Belgium, as well as by the National Ethics Council for Research in Health (CNERS) in Dakar, Senegal (on 24 April 2019, Ref. SEN19/08).

### Reporting summary

Further information on research design is available in the Nature Portfolio Reporting Summary linked to this article.

## Online content

Any methods, additional references, Nature Portfolio reporting summaries, source data, extended data, supplementary information, acknowledgements, peer review information; details of author contributions and competing interests; and statements of data and code availability are available at 10.1038/s41591-023-02719-4.

### Supplementary information

Supplementary Information Supplementary Tables 1 and 2.

Reporting Summary

## Data Availability

Open access information on the SchistoSAM trial such as the trial protocol, informed consent form and statistical analysis plan is published in the Clinicaltrials.gov registry with code NCT03893097. These documents, as well as the REDCap codebook and statistical codes have been publicly available in the Zenodo repository (at https://zenodo.org/records/10089112; 10.5281/zenodo.10089112). Individual participant data have been deposited in Zenodo also, but is subject to controlled access due to privacy reasons and the European data protection legislation and can only be accessed after review and approval by the Data Access Committee (DAC) of the Institute of Tropical Medicine (full information at https://www.itg.be/en/research/data-sharing-and-open-access). A request can be made by submitting a data access request form to ITMresearchdataaccess@itg.be/. The DAC will review the suitability of the data for the secondary research proposal, the amount of (indirect) identifiers needed and the possibility of sufficient anonymization, as well as the scientific value and ethical aspects, in collaboration with the principal investigators. An answer to the requests will be formulated within a month. The DAC has the expertise to anonymize the data (in line also with the European General Data Protection Regulation) and will draw up a data sharing agreement before data can be shared.

## Code availability

In line with the previous statement, statistical codes have been made available in the Zenodo repository (see above for identifiers).
