# Supplementary material for: Antimalarial artesunate–mefloquine versus praziquantel in African children with schistosomiasis: an open-label, randomized controlled trial
Source: Nat Med. 2024 Jan 4;30(1):130–7. doi: 10.1038/s41591-023-02719-4 (PMC10803269; doi:10.1038/s41591-023-02719-4)
Supplement: Supplementary file 1 — Supplementary Tables 1 and 2. [file 41591_2023_2719_MOESM1_ESM.pdf]

nature medicine

Article https://doi.org/10.1038/s41591-023-02719-4

Antimalarial artesunate–mefloquine versus praziquantel in African children with schistosomiasis: an open-label, randomized controlled trial

In the format provided by the authors and unedited

Supplementary Table 1: Sensitivity analyses of cure rate in the artesunate-mefloquine arm versus the praziquantel arm at week 4 post-treatment, in the intention-to-treat (ITT) population

A. Intention-to-treat analysis: on any available sample result

All subjects who were randomized were included in the ITT analysis populations. In cases where one of the two urine results, or any of the four stool results were missing, then the schistosomiasis status calculations were based only on the available samples.

Species	Artesunate-mefloquine	Praziquantel	Cure rate difference (95% CI)
Both species pooled			
Cure rate n/n (%; 95% CI)	211 / 357 (59.1%; 53.9-64.1)	215 / 351 (61.3%; 56.1-66.2)	-2.1% (-9.3 – 5.0)
S. haematobium			
Cure rate n/n (%; 95% CI)	212 / 352 (60.2%; 55.0-65.2)	217 / 351 (61.8%; 56.6-66.8)	-1.6% (-8.8 – 5.6)
S. mansoni			
Cure rate n/n (%; 95% CI)	48 / 54 (88.9%; 77.8-94.8)	52 / 54 (96.3%; 87.5-99.0)	-7.4% (-18.8 – 3.2)

B. Intention-to-treat analysis: on complete samples only

All randomized subjects were included in this analysis, excluding those that had any missing microscopy results.

Species	Artesunate-mefloquine	Praziquantel	Cure rate difference (95% CI)
Both species pooled			
Cure rate n/n (%; 95% CI)	211 / 357 (59.1%; 53.9-64.1)	214 / 350 (61.1%; 55.9-66.1)	-2.0% (-9.2 – 5.2)
S. haematobium			
Cure rate n/n (%; 95% CI)	212 / 352 (60.2%; 55.0-65.2)	216 / 350 (61.7%; 56.5-66.7)	-1.5% (-8.7 – 5.7)
S. mansoni			
Cure rate n/n (%; 95% CI)	48 / 54 (88.9%; 77.8-94.8)	51 / 53 (96.2%; 87.2-99.0)	-7.3% (-18.8 – 3.4)

C. Intention-to-treat analysis: best case scenario

All missing samples were considered as negative in the calculations of the cure rate.

Species	Artesunate-mefloquine	Praziquantel	Cure rate difference (95% CI)
Both species pooled			
Cure rate n/n (%; 95% CI)	214 / 360 (59.4%; 54.3-64.4)	221 / 357 (61.9%; 56.8-66.8)	-2.5% (-9.6 - 4.7)
S. haematobium			
Cure rate n/n (%; 95% CI)	215 / 355 (60.6%; 55.4-65.5)	223 / 357 (62.5%; 57.3-67.3)	-1.9% (-9.0 – 5.2)
S. mansoni			
Cure rate n/n (%; 95% CI)	48 / 54 (88.9%; 77.8-94.8)	53 / 55 (96.4%; 87.7-99.0)	-7.5% (-18.9 – 3.0)

D. Intention-to-treat analysis: worst case scenario

All missing samples were considered as positive in the calculations of the cure rate.

Species	Artesunate-mefloquine	Praziquantel	Cure rate difference (95% CI)
Both species pooled			
Cure rate n/n (%; 95% CI)	211 / 360 (58.6%; 53.5-63.6)	215 / 358 (60.1%; 54.9-65.0)	-1.4% (-8.6 – 5.7)
S. haematobium			
Cure rate n/n (%; 95% CI)	212 / 355 (59.7%; 54.5-64.7)	217 / 358 (60.6%; 55.5-65.5)	-0.9% (-8.0 – 6.3)
S. mansoni			
Cure rate n/n (%; 95% CI)	50 / 56 (89.3%; 78.5-95.0)	53 / 57 (93.0%; 83.3-97.2)	-3.7% (-15.3 – 7.5)

Supplementary Table 2: Cure rates, using microscopic results, and cure rate difference and cure rate ratios between artesunate-mefloquine and praziquantel arms at the different study time points, per *Schistosoma* species and with both pooled species

Species		Artesunate-mefloquine<br>Cured (cure rate; 95% CI)	Praziquantel<br>Cured (cure rate; 95% CI)	Cure rate difference<br>(95% CI)	Cure rate ratios<br>(95% CI)
S. haematobium	Week 4	209 / 344 (0.608; 0.555-0.658)	213 / 340 (0.626; 0.574-0.676)	-0.019 (-0.091- 0.054)	0.97 (0.86-1.09)
	Week 10	263 / 339 (0.776; 0.728-0.817)	258 / 335 (0.770; 0.722-0.812)	0.006 (-0.058- 0.069)	1.01 (0.93-1.09)
	Week 16	294 / 336 (0.875; 0.835-0.906)	263 / 333 (0.790; 0.743-0.830)	0.085 (0.029- 0.142)	1.11 (1.03-1.19)
	Week 24	274 / 318 (0.862; 0.819-0.895)	253 / 325 (0.778; 0.730-0.820)	0.083 (0.024- 0.142)	1.11 (1.03-1.19)
	Week 48	58 / 290 (0.200; 0.158-0.250)	50 / 304 (0.164; 0.127-0.210)	0.036 (-0.027- 0.098)	1.22 (0.86-1.71)
S. mansoni	Week 4	48 / 54 (0.889; 0.778-0.948)	50 / 52 (0.962; 0.870-0.989)	-0.073 (-0.187- 0.036)	0.92 (0.83-1.03)
	Week 10	49 / 53 (0.925; 0.821-0.970)	50 / 52 (0.962; 0.870-0.989)	-0.037 (-0.144- 0.065)	0.96 (0.87-1.06)
	Week 16	51 / 52 (0.981; 0.899-0.997)	51 / 52 (0.981; 0.899-0.997)	0.000 (-0.083- 0.083)	1.0 (0.95-1.05)

	Week 24	44 / 51 (0.863; 0.743-0.932)	47 / 52 (0.904; 0.794-0.958)	-0.041 (-0.173- 0.089)	0.95 (0.83-1.1)
	Week 48	28 / 48 (0.583; 0.443-0.712)	33 / 50 (0.660; 0.522-0.776)	-0.077 (-0.259- 0.112)	0.88 (0.64-1.19)
Pooled	Week 4	208 / 349 (0.596; 0.544-0.646)	211 / 340 (0.621; 0.568-0.671)	-0.025 (-0.097- 0.048)	0.96 (0.85 - 1.08)
	Week 10	262 / 343 (0.764; 0.716-0.806)	255 / 335 (0.761; 0.713-0.804)	0.003 (-0.061- 0.067)	1.0 (0.92 - 1.09)
	Week 16	297 / 340 (0.874; 0.834-0.905)	261 / 333 (0.784; 0.736-0.825)	0.090 (0.033- 0.146)	1.11 (1.04 - 1.19)
	Week 24	270 / 322 (0.839; 0.794-0.875)	246 / 325 (0.757; 0.707-0.800)	0.082 (0.020- 0.143)	1.11 (1.02 - 1.2)
	Week 48	54 / 294 (0.184; 0.144-0.232)	48 / 304 (0.158; 0.121-0.203)	0.026 (-0.035- 0.086)	1.16 (0.82-1.66)

Note: CI denotes confidence interval; The ratios of cure rates were calculated per time point together with the Wald confidence interval.
