# Supplementary material for: Antimalarial artesunate–mefloquine versus praziquantel in African children with schistosomiasis: an open-label, randomized controlled trial
Source: Nat Med. 2024 Jan 4;30(1):130–7. doi: 10.1038/s41591-023-02719-4 (PMC10803269; doi:10.1038/s41591-023-02719-4)
Supplement: Supplementary file 2 — Reporting Summary [file 41591_2023_2719_MOESM2_ESM.pdf]

nature portfolio

Corresponding author(s): BOTTIEAU E; NMED-A125703B

Last updated by author(s): Nov 8, 2023

Reporting Summary

Nature Portfolio wishes to improve the reproducibility of the work that we publish. This form provides structure for consistency and transparency in reporting. For further information on Nature Portfolio policies, see our Editorial Policies and the Editorial Policy Checklist.

Statistics

For all statistical analyses, confirm that the following items are present in the figure legend, table legend, main text, or Methods section.

n/a	Confirmed	
☐	☒	The exact sample size (*n*) for each experimental group/condition, given as a discrete number and unit of measurement
☐	☒	A statement on whether measurements were taken from distinct samples or whether the same sample was measured repeatedly
☐	☒	The statistical test(s) used AND whether they are one- or two-sided<br>*Only common tests should be described solely by name; describe more complex techniques in the Methods section.*
☐	☒	A description of all covariates tested
☒	☐	A description of any assumptions or corrections, such as tests of normality and adjustment for multiple comparisons
☐	☒	A full description of the statistical parameters including central tendency (e.g. means) or other basic estimates (e.g. regression coefficient) AND variation (e.g. standard deviation) or associated estimates of uncertainty (e.g. confidence intervals)
☒	☐	For null hypothesis testing, the test statistic (e.g. *F*, *t*, *r*) with confidence intervals, effect sizes, degrees of freedom and *P* value noted<br>*Give P values as exact values whenever suitable.*
☒	☐	For Bayesian analysis, information on the choice of priors and Markov chain Monte Carlo settings
☒	☐	For hierarchical and complex designs, identification of the appropriate level for tests and full reporting of outcomes
☒	☐	Estimates of effect sizes (e.g. Cohen's *d*, Pearson's *r*), indicating how they were calculated

Our web collection on statistics for biologists contains articles on many of the points above.

Software and code

Policy information about availability of computer code

Data collection	For data collection, Research Electronic Data Capture (REDCap) v8.10.4 was used
Data analysis	SAS v9.4 and R v4.2.2 were used for the statistical analyses

For manuscripts utilizing custom algorithms or software that are central to the research but not yet described in published literature, software must be made available to editors and reviewers. We strongly encourage code deposition in a community repository (e.g. GitHub). See the Nature Portfolio guidelines for submitting code & software for further information.

Data

Policy information about availability of data

All manuscripts must include a data availability statement. This statement should provide the following information, where applicable:

- Accession codes, unique identifiers, or web links for publicly available datasets
- A description of any restrictions on data availability
- For clinical datasets or third party data, please ensure that the statement adheres to our policy

A set of study-related documents such as the study protocol, statistical analysis plan, inform consent form, CONSORT checklist,... have been made publicly available. in Zenodo repository (at https://zenodo.org/records/10082977), with the following DOI 10.5281/zenodo.10082977). De-identified individual participant data and a data dictionary defining each field in the set can be made available to others after publication, wit a controlled access (for pricon approval of a written request to

the Data Access Committee of the Institute of Tropical Medicine, Antwerp (mail to at . The request will be evaluated for suitability and scientific value of the secondary research by this Data Access Committee, in collaboration with the principal investigators. A data sharing agreement will be needed.

Human research participants

Policy information about studies involving human research participants and Sex and Gender in Research.

Reporting on sex and gender	Only gender was collected in this study, based .on parents' reporting or self-reporting (depending on children's age)
Population characteristics	Primary school-aged children (6-14 years old) from six selected villages in Senegal, who tested positive for schistosomiasis
Recruitment	The selection of the six endemic villages was based on a parasitological survey prior to the trial. The purpose and methods of the trial were presented in detail to the community leaders, school directors, teaching staff and children's parents. Written informed consent for schistosomiasis screening and trial participation upon positivity, was asked from the children's parents or legal guardians. Oral assent was also obtained from the children.<br>Enrollment to the trial was offered to the children found with Schistosoma eggs by microcopic examination of urine and/or stool. found e found positive. The main bias was that only school-attending children were recruited. Other children in the communities (not attending school) might have had higher intensities of infection, and possibly lower response to the evaluated drugs.
Ethics oversight	The trial was approved by the Institutional Review Board of the Institute of Tropical Medicine (on 30 January 2019, Ref. 1269/18) and the Ethics Committee of the University of Antwerp, (on 21 January 2019, Ref. 19/02/005), in Antwerp, Belgium, as well as by the National Ethics Council for Research in Health (CNERS) in Dakar, Senegal (on 24 April 2019, Ref. SEN19/08).

Note that full information on the approval of the study protocol must also be provided in the manuscript.

Field-specific reporting

Please select the one below that is the best fit for your research. If you are not sure, read the appropriate sections before making your selection.

☒ Life sciences ☐ Behavioural & social sciences ☐ Ecological, evolutionary & environmental sciences

For a reference copy of the document with all sections, see nature.com/documents/nr-reporting-summary-flat.pdf

Life sciences study design

All studies must disclose on these points even when the disclosure is negative.

Sample size	The sample size was calculated by simulation, for the non-inferiority of AM versus PZQ. A cure rate of 75% was assumed for both treatments with a non-inferiority margin of 10%. To ensure a power of 80% a sample size of 300 participants per arm would be needed.The sample size was corrected for an expected 20% attrition, thus increasing the final sample size to 360 participants per arm.
Data exclusions	Participants who were found negative for schistosomiasis were excluded from all analyses and were not randomized according to the study exclusion criteria. Participants with no available samples at baseline were also excluded. A further 5 participants were excluded from all analyses due to suspicion of misidentification. For efficacy analyses, both an intention to treat and per-protocol analyses populations were defined. In the per-protocol population, all participants that did not follow the study procedures according to the protocol (exclusion criteria, erroneous treatment, missed treatment, no stool/urine samples, dosage miscalculation, out-of-window visit) were excluded. The per-protocol population was the primary analysis population as recommended for non-inferiority trials.
Replication	No explicit measures for replication were taken in this study, considering the unique setting and timing the trial was set. However, a detailed description of sampling, laboratory procedures and statistical analyses would allow a similar experiment to be conducted.
Randomization	Blocked randomization, stratified by Schistosoma species was used to randomize participants into the two treatment arms. Opaque sealed envelopes were used to ensure that the randomization process will be respected
Blinding	This was an open-label clinical trial, thus both participants and investigators were unblinded to the allocated treatment. Microscopists who assessed the parasitological efficacy (urine/stool) were blinded to treatment arms.

Reporting for specific materials, systems and methods

We require information from authors about some types of materials, experimental systems and methods used in many studies. Here, indicate whether each material, system or method listed is relevant to your study. If you are not sure if a list item applies to your research, read the appropriate section before selecting a response.

Materials & experimental systems

n/a	Involved in the study
☒	☐ Antibodies
☒	☐ Eukaryotic cell lines
☒	☐ Palaeontology and archaeology
☒	☐ Animals and other organisms
☐	☒ Clinical data
☒	☐ Dual use research of concern

Methods

n/a	Involved in the study
☒	☐ ChIP-seq
☒	☐ Flow cytometry
☒	☐ MRI-based neuroimaging

Clinical data

Policy information about clinical studies

All manuscripts should comply with the ICMJE guidelines for publication of clinical research and a completed CONSORT checklist must be included with all submissions.

Clinical trial registration	NCT03893097
Study protocol	DOI: 10.1136/bmjopen-2020-047147
Data collection	Clinical data were collected via the REDCap tool directly in the study sites (primary schools of the selected villages in Richard Toll district, Senegal. Results of lthe aboratory analyses performed in Richard Toll hospital, in Dakar (IRESSEF) and in Antwerp (ITM) were also collected via REDCap.
Outcomes	The primary objectives of this noninferiority trial were to compare the cure rate (as assessed by measurement of egg excretion in urine/stool by microscopy) and safety (frequency and type of adverse events) of a three-day course of the artesunate-mefloquine combination at antimalarial dosage versus the standard-care single-dose praziquantel for the treatment of schistosomiasis in African primary school-aged children. The efficacy outcome (cure rate) was measured by the proportion of trial participants without any Schistosoma eggs at Week 4 (assessed by urine filtration and duplicate Kato-Katz). The safety outcome was measured by the proportion of participants reporting any adverse events during and after the study drug administration during field visiti). Relatedness with the drug was determined by the local clinical investigators.<br>The secondary objectives were (1) to determine the egg reduction rates by Schistosoma species (as assessed by comparing the egg concentrations for each species by microscopy in urine and stool before and at Week 4 after treatment); (2) to assess the cumulative antischistosomal efficacy and toxicity of one and two additional courses of artesunate-mefloquine (measured in a similar way as for the primary outcomes, but at week 10 and 16 (i.e. 4 weeks after each additional course of artesunate-mefloquine), and (3) to monitor the incidence of clinical malaria (by capturing any clinical malaria diagnosed among the participants at the health facilities of the selected villages during the study period) and explore P. falciparum malaria infection and possible emergence of resistant markers (assessed by the proportion of (asymptomatic) malaria infection diagnosed in trial participants by molecular methods at inclusion and at the 6-month assessment, as well as the presence and frequency of markers of parasite resistance).
